# Ionic Liquid-Based Microwave-Assisted Extraction of Flavonoids from *Bauhinia championii (Benth.) Benth*.

**DOI:** 10.3390/molecules171214323

**Published:** 2012-12-03

**Authors:** Wei Xu, Kedan Chu, Huang Li, Yuqin Zhang, Haiyin Zheng, Ruilan Chen, Lidian Chen

**Affiliations:** 1 Pharmacy College of Fujian University of Traditional Chinese Medicine, Fuzhou 350122, China; 2 Integrative Medicine College of Fujian University of Traditional Chinese Medicine, Fuzhou 350122, China; 3 Rehabilitation Medicine College of Fujian University of Traditional Chinese Medicine, Fuzhou 350122, China

**Keywords:** ionic liquid, *Bauhinia championii (Benth.) Benth.*, flavonoids, LC-MS

## Abstract

An ionic liquids (IL)-based microwave-assisted approach for extraction and determination of flavonoids from *Bauhinia championii (Benth.) Benth*. was proposed for the first time. Several ILs with different cations and anions and the microwave-assisted extraction (MAE) conditions, including sample particle size, extraction time and liquid-solid ratio, were investigated. Two M 1-butyl-3-methylimidazolium bromide ([bmim] Br) solution with 0.80 M HCl was selected as the optimal solvent. Meanwhile the optimized conditions a ratio of liquid to material of 30:1, and the extraction for 10 min at 70 °C. Compared with conventional heat-reflux extraction (CHRE) and the regular MAE, IL-MAE exhibited a higher extraction yield and shorter extraction time (from 1.5 h to 10 min). The optimized extraction samples were analysed by LC-MS/MS. IL extracts of *Bauhinia championii (Benth.) Benth* consisted mainly of flavonoids, among which myricetin, quercetin and kaempferol, β-sitosterol, triacontane and hexacontane were identified. The study indicated that IL-MAE was an efficient and rapid method with simple sample preparation. LC-MS/MS was also used to determine the chemical composition of the ethyl acetate/MAE extract of *Bauhinia championii (Benth.) Benth*, and it maybe become a rapid method to determine the composition of new plant extracts.

## Abbreviations

IL: ionic liquid
MAE: microwave-assisted extraction
CHRE: conventional heat-reflux extraction

## 1. Introduction

*Bauhinia championii (Benth.)Benth.*, first recorded in the book *Nanning Drug Chi*, belongs to the bauhinia leguminosae family. It is bitter and acerbic in taste, mild in nature. It has the ability of expelling wind to eliminate dampness, promoting blood circulation to stop pain, invigorating the spleen and regulating qi [1]. Its effects are recorded in many books, such as *The National Assembly of Chinese Herbal Medicine*, *Dictionary of Chinese Traditional Medicine* and *The Chinese Herbal*. In folk medicine, it is mainly used to treat epigastric pain [2], rheumatic arthritis [3], acute and chronic lumbar and leg pain [4,5]. According to the records in *China Shezu Medicine* and investigations of medical statistics among the She ethnic minority group, many doctors prescribed this medicine for rheumatic pain*.* It could improve the symptomatic signs of RA, obviously reduce arthragia and swelling and had good clinical effects. Contemporary pharmacological studies have shown that *Bauhinia championii (Benth.) Benth.* displays pharmacological effects and biocological activities in many areas, such as anti-platelet aggregation [6], anti-inflammatory and analgesia [7], anti-infection [8]. The literature indicates that it contains flavonoids, sterols, aromatic acids, terpenoids and so on [9,10,11,12]. In recent years, with the increase of rheumatism and rheumatic related morbidity, research of *Bauhinia championii (Benth.) Benth.* has increasingly received more attention.

Recently, a variety of developed extraction techniques have played a key role in separation and analysis. Compared with other modern extraction techniques such as supercritical fluid extraction, microwave-assisted extraction (MAE) technology has interesting features such as less extraction time, energy savings, lower dosage of solvents, fast heating speed, high efficiency, and so on. It has been applied widely to extract natural products [13,14,15], but the extracting solvents used often include organic solvents, which have strong volatility and toxicity and can cause serious pollution issues. Ionic liquids (ILs) are composed of organic cations and inorganic or organic anions, and are liquid at or near room temperature (or conventionally below 100 °C). They are known as “green” solvents for their many advantages as excellent chemical and thermal stability, low vapor pressure, non-volatility, non-combustibility, highly conductivity, wide liquid processability and designability. In recent years, ILs have received much attention as neoteric solvents in various applications including catalysis, synthesis, industrial cleaning, extraction and separation [16,17,18,19,20]. The potential value in research of ionic liquids has been recognized by chemists worldwide. It has potential to bring immense progress to modern industry, faced with important problems such as pollution and safety. The extraction technique that combines microwave extraction technology with ionic liquids for application in the field of extraction and separation constitutes a new approach. Recently, ionic liquids were investigated as solvents in the extraction of *trans*-resveratrol from Rhizma Polygoni Cuspidati [21], polyphenolic compounds [22] and alkaloids from medicinal plants [23,24]. But to our best knowledge, until now, ILs-MAE has not yet been reported for extraction of flavonoid compounds from *Bauhinia championii (Benth.) Benth*. 

This study investigated the potentiality of various ILs with different cations and anions as microwave absorption media, as well as the microwave parameters of an IL-MAE method which was used to extract flavonoid from *Bauhinia championii (Benth.) Benth*. LC-MS was performed to analyze the chemical composition of *Bauhinia championii (Benth.)Benth. *The aim of this work was to develop an effective, rapid, validated and environmentally friendly IL-MAE approach for extraction from this herbal medicine, and analyse the chemical composition of the extraction of *Bauhinia championii (Benth.) Benth*.

## 2. Results and Discussion

### 2.1. Compositions of Bauhinia championii (Benth.) Benth

**Figure 1:**
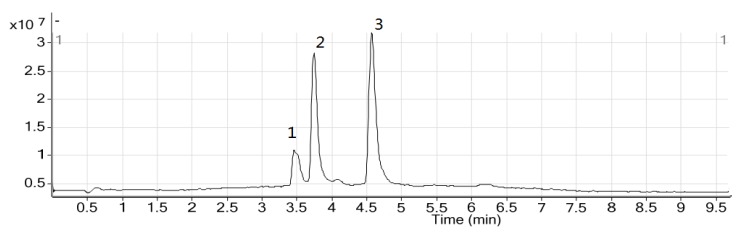
The total ion chromatogram of sample: (**1**) myricetin; (**2**) quercetin; (**3**) kaempferol.

The obtained extract from the IL-MAE approach was analysed by LC-MS-MS. The negative ion mode is used for detection of the total flavonoid compound with many hydroxy groups, which can generate a stable oxygen anion. It makes the total ion chromatogram have lower background values and the parent ion is normally [M−H]^−^, which is easily recognized. LC-MS-MS has been successfully applied for a quick separation and identification of the major components of *Bauhinia championii (Benth.) Benth*. The total ion chromatograms of a sample are shown in Figure 1. The fragment pattern *m/z *301, 317, 285 was found in its first order mass spectrum, and it is speculated that they may correspond to the fragment patterns of myricetin, quercetin and kaempferol, respectively. Analysis of the three reference substances, myricetin, quercetin and kaempferol, was done under the same conditions to prove the inference. Fragment ions were quercetin (*m/z *121, 151, 179, 245, 273, 301), myricetin (*m/z *137, 151, 179, 271, 289, 299, 317), kaempferol (*m/z *187.45, 92.85, 285.02). Consideration of both their architectural features and their fragment ions, indicated that the fragmentation of the three reference substances were same as that of the extracts (*m/z *301, 317 and 285). Comparison to the reference substance and a mass spectral library search system, confirmed the presence of components such as myricetin, quercetin, kaempferol, β-sitosterol, triacontane, and hexacontane. 

### 2.2. HPLC Analysis of Sample and Quantification

**Figure 2:**
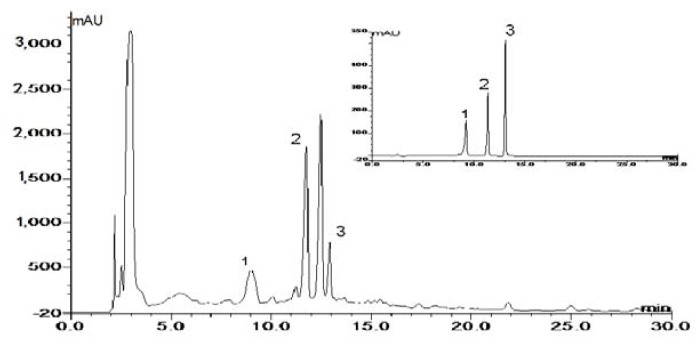
Examples of chromatograms of standards and sample: (**1**) myricetin; (**2**) quercetin; (**3**) kaempferol, the inset shows the chromatogram of three standard substances.

The three flavonols were identified by their corresponding chromatograms, spectrograms and retention times in comparison with authentic standard compounds. Examples of chromatograms of standards and sample are shown in Figure 2. It was no effects attributable to the ILs that were observed on elution order, elution times and peak resolution and so on. The yields of myricetin, quercetina and kaempferol below were quantified by this method.

### 2.3. Screening of ILs

ILs have strong polarity and a special capacity for organic and inorganic dissolvability due to their considerable coulomb force. Also they can efficiently absorb microwave energy, thus were used as solvents and microwave absorption mediums in the MAE [25,26]. The structure of ILs has significant effect on its physicochemical properties and the extraction yields, because of their multiple interactions with analytes [27] and dissolving ability for polyphenolic compounds [28]. In this study, various ILs with different cations and anions aqueous solutions were studied at conditions of extraction time: 10 min, particle size: 0.30–0.45 mm, S/L ratio: 1:30, HCl: 0.8 mol/L and concentration of each ILs: 1.5 M. The ILs and the results of the yields of myricetin, quercetina and kaempferol are shown in Table 1.

**Table 1:** ILs’ effects on yields of myricetin, quercetina and kaempferol.

Solvent	Yield (mean ± SD, mg/g)		
	Myricetin	Quercetin	Kaempferol
[bmim] Br	0.0497 ± 0.02	0.2375 ± 0.02	0.0305 ± 0.03
[bmim] Cl	0.0103 ± 0.01	0.1257 ± 0.04	0.0211 ± 0.01
[bmim] [PF_6_]	0.0163 ± 0.02	0.1078 ± 0.01	0.0718 ± 0.01
[bmim] [BF_4_]	0.0411 ± 0.03	0.1065 ± 0.01	0.0190 ± 0.02
[bmim] [H_2_PO_4_]	0.0194 ± 0.03	0.1035 ± 0.03	0.0289 ± 0.02
[bmim]_2 _[SO_4_]	0.0110 ± 0.01	0.0953 ± 0.05	0.0114 ± 0.04
[bmim] [HSO_4_]	0.0200 ± 0.01	0.1453 ± 0.05	0.0274 ± 0.04
[hmim] Br	0.0127 ± 0.01	0.1329 ± 0.03	0.0181 ± 0.03
[emim] Br	0.0211 ± 0.03	0.1556 ± 0.01	0.0390 ± 0.02
[HOOCCH_2_-mim] Cl	0.0174 ± 0.01	0.1375 ± 0.02	0.0225 ± 0.01

Note: [bmim] is 1-butyl -3-methylimidazolium; [emim] is 1-ethyl -3-methylimidazolium; [hmim] is 1-hexyl -3-methylimidazolium; HOOCCH_2_-mim is 1- HOOCCH_2_ -3-methylimidazolium.

The identity of the anion in an IL has a strong influence on its physicochemical properties [29], such as the water miscibility. Therefore, 1-butyl-3-methylimidazolium ILs were investigated with several different anions (Cl^−^, Br^−^, BF_4_^−^, PF_6_^−^, H_2_PO_4_^−^, SO_4_^2−^, HSO_4_^−^). The extraction yield was accordingly different (see Table 1). ILs with the anions Cl^−^, Br^−^and BF_4_^−^ are hydrophilic and miscible in any proportion with water; however ILs with anions PF_6_^−^ are hydrophobic and only partly soluble in water. [bmim] [PF_6_] showed low extraction efficiency, which might be due to its hydrophobicity and was only sparingly water soluble. The reason for the differences of extraction yield among Cl^−^, Br^−^, BF_4_^−^ maybe due to the fact that Br^−^ had stronger solvation power and multi-interactions including hydrogen bonding, polarity, ionic/charge–charge and π-π, π-n with flavonoid and hydrolyzed the flavonol glycosides [30], all of which contribute to increase the solubility of flavonoids. In consideration of evaluating the yield of myricetin, quercetin and kaempferol at the same time, the Br^−^ anion was selected for futher study. 

Aqueous solutions of three species of 1-alkyl-3-methylimidazolium cations with the same Br^−^anion ([emim] Br, [bmim] Br and [hmim] Br) were employed to research the effects of the alkyl chain length on the extraction yield. The results suggested that the increasing alkyl chain length did influence the extraction yield, and [bmim] Br was more efficient than the other two. Proton acidity for the three cations increased from ethyl to hexyl at the 1-position of the 1-alkyl-3-methylimidalizium ring [28], but the hydrophobicity also increased with the increase of the alkyl chain length. Both the proper hydrogen bonding and hydrophobic interactions of [bmim] Br resulted in stronger solvation interactions with flavonoids followed by higher extraction yields than those of [emim] Br and [hmim] Br.

The other ILs were used as dual catalyst solvents in the hydrolysis of flavonol glycosides; [bmim] [H_2_PO_4_] is a weak Brönsted acidic ionic liquid. The H^+^ concentration was low so that the [bmim] [H_2_PO_4_] solution did not effectively hydrolyze the flavonol glycosides, but [bmim] [HSO_4_] and [HOOCCH_2_mim] Cl which could provide enough H^+^ to hydrolyze the flavonol glycosides showed strong hydrolysis ability, resulting in high yields. Considering that the hydrolysis ability of acidified IL solutions [31] and the extraction ability of [bmim]Br for flavonols (see Table 1), acidified [bmim]Br solution was selected in order to investigate the hydrolysis ability of acidified ILs for the flavonol glycosides.

**Figure 3:**
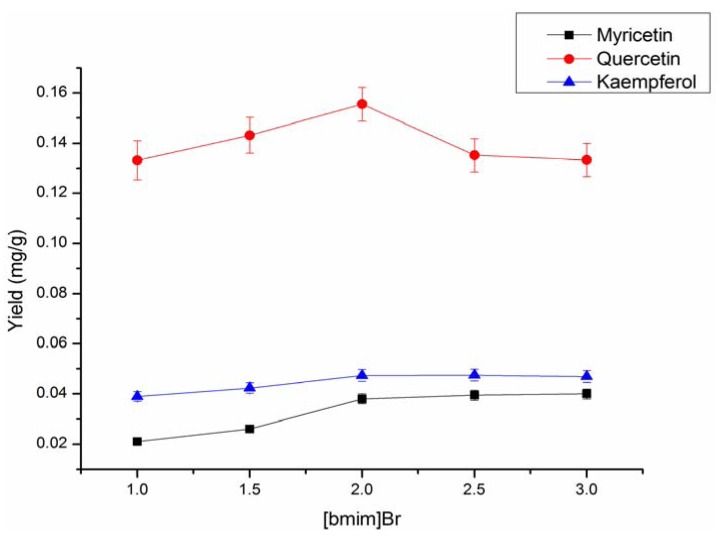
Effect of [bmim] Br concentrations on flavonoids extraction yields (Particle size: 0.30 to 0.45 mm; S/L ratio: 1:20; HCl: 0.8 mol/L). The range of [bmim] Br concentration was from 1.5 to 3.0 mol/L.

Moreover, the optimum concentration of acidified [bmim]Br was also studied. IL was only used as a solvent and additive to enhance the hydrolysis of flavonol glycosides. HCl concentration played a very important role in the hydrolysis of flavonol glycosides [32], so the presence of HCl in [bmim] Br was necessary for the hydrolysis of the myricetin, quercetin and kaempferol glycosides. According to the results of Wang *et al*. [33] and some of our primary studies, a concentration of 0.80 mol/L HCl in the [bmim]Br solution was enough for extraction. Figure 3 shows that the extraction yields were concentration-dependent. The reasons relate to the solubility of flavonoids and hydrolysis of the flavonol glycosides in the extraction solvents. However, when the concentration was increased to a certain extent, the extraction yields increased slowly, which was due to the diffusion and transfer capacity changing slightly and even the solvation ability increased.

From the results in Figure 3, the extraction efficiency increased with IL concentration in the range of 1.0–2.0 M. When it increased further, however, a decrease was observed, so finally 2.0 M [bmim]Br with 0.80 mol/L HCl was selected as the optimum medium.

### 2.4. Optimization of ILs-MAE Extraction Conditions

Many factors affect the extraction yield obtained by IL-MAE. Based on various literature reports and the preliminary experiments carried out, the main extraction parameters (sample particle size, extraction time, solid-liquid ratio and eatraction temperature) were selected to optimize the IL-MAE extraction conditions by the univariate method.

**Figure 4:**
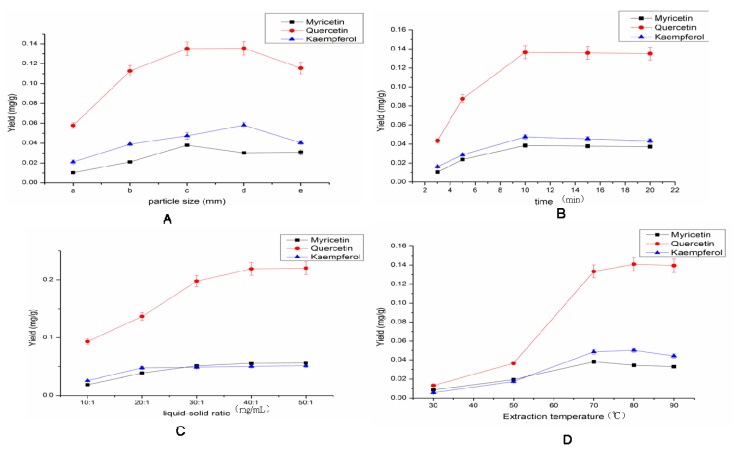
Effect of different factors on flavonoid extraction yields. (**A**) Effect of different particle size on flavonoids extraction yields (extraction time: 10 min; S/L ratio: 1:20; extraction temperature: 70 °C; HCl: 0.8 mol/L). (**B**) Effect of different time on flavonoids extraction yields (particle size : 0.30 to 0.45 mm; S/L ratio: 1:20; extraction temperature: 70 °C; HCl: 0.8 mol/L). (**C**) Effect of different S/L ratio on flavonoid extraction yields (extraction time: 10 min; particle size: 0.30 to 0.45 mm; extraction temperature: 70 °C; HCl: 0.8 mol/L). (**D**) Effect of different extraction temperatures on flavonoid extraction yields (S/L ratio: 1:20; extraction time: 10 min; particle size: 0.30 to 0.45 mm; HCl: 0.8 mol/L).

As shown in Figure 4A, the particle size of the powders had a remarkable effect on the extraction yield, which increased with particle size of the powders from a (2 mm to 1.19 mm) to c (0.5 mm to 0.35 mm), however it did not change much or even decreased when the particle size was smaller. It may be because that ILs are liquids at room temperature or close to room temperature, and have weak diffusion capacity, so it was difficult to penetrate into the sample, therefore flavonoids cannot dissolve completely and quickly. When the size was too small, the sample conglomerated, it was also difficult to penetrate into the sample. Herein a particle size ranged from 0.30 mm to 0.45 mm was therefore selected.

As shown in Figure 4B the yield of extraction significantly increased with extended time within 3~10 min. When the extraction time exceeded 10 min, the yield of extraction remained almost the same. It may be that there will always be a microwave-heating process. The dissolution rate of flavonoids was mainly subject to heat within 3~10 min. Therefore, it varied inversely with the temperature. When the time reached 10 min, the majority of plant cell wall were disrupted. At that moment, the dissolution rate was mainly affected by diffusion speed. Ten minutes was therefore chosen as the extraction time based on diffusion speed and the flavonoids’ concentration increased in the extraction.

As for the liquid–solid ratio, Figure 4C shows that the yields increased rapidly when the ratio was changed from 10:1 to 40:1. Between 30:1 and 40:1, the yield of myricetin, quercetin and kaempferol was stable. Taking also the considerations of cost and reactions conditions into account, a liquid–solid ratio of 30:1 was selected in this work.

Figure 4D shows that the extraction yields increased with the increase in temperature in the range of 30–70 °C. From 70 to 90 °C, the extraction yields of the three flavonols decreased slightly. The increasing extractiin temperature was conducive to reducing the viscosity and enhancing the spreadability and solubility of ILs, which was good to dissolve the flavonols, but when the temperature reached 70 °C, the sample became black, indicating a probable degradation of the flavonols. Herein the extraction temperature of 70 °C was selected. 

### 2.5. Comparison of the Proposed ILs-MAE Approach with the Other Conventional Extraction Methods

**Figure 5:**
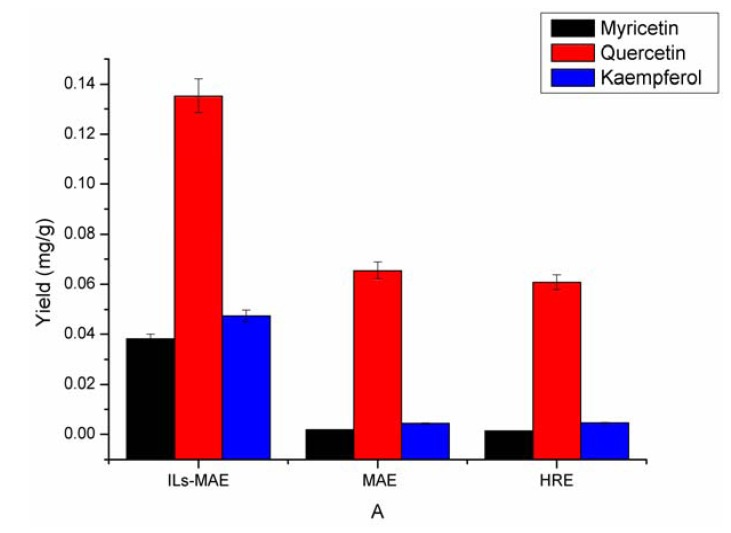
The extraction yields using different extraction methods (extraction time: 10 min; S/L ratio: 1:30; particle size: 0.30 to 0.45 mm; HCl: 0.8 mol/L).

A comparison of the proposed IL-MAE approach to extract myricetin, quercetin and kaempferol from *Bauhinia championii (Benth.) Benth* with other conventional extraction methods such as HRE and regular MAE extraction method are carried out*.* Based on the results of Figure 5, the extraction yields were in the order IL-MAE > MAE > CHRE. IL-MAE not only obviously increased the extraction yield, but also reduced the extraction time and saved organic solvent. The diverse results among the three methods were mainly due to the particular extraction mechanisms. Microwave heating involves superheating, mass heating and fast heating, HRE does not. Molecular motion could be even more intense, so the ingredients dissolved more efficiently. IL-MAE had both the advantages of MAE and IL. It can be concluded that the proposed ILs-MAE approach was a rapid and effective approach for extracting myricetin, quercetin and kaempferol from *Bauhinia *championii (Benth.) Benth.

### 2.6. Method Validation

To demonstrate the proposed IL-MAE approach, validation tests were investigated. Parameters such as linearity, precision, reproducibility, average recovery and stability were determined under the optimized conditions.

Table 2 shows that the linearity was good, with a regression coefficient greater than 0.999. The reproducibility study was carried out by determining five repetitive standards. The results indicated it had good reproducibility; its RSD obtained was 1.13%, 1.56%, 1.21%. The recoveries were in the range of 94.6% and 105.5%, and the RSD was lower than 5%. Precision and stability were also investigated, confirming that the proposed IL-MAE approach was bothstable and reliable.

**Table 2:** Calibration curves of the proposed method.

Analytes	Linearities	Correlation coefficients	Calibration range (μg·mL^−1^)
Myricetin	Y = 0.583x − 0.5086	R^2^ = 0.9996	X = 30–110
Quercetin	Y = 0.7856x − 21.681	R^2^ = 0.9998	X = 80–300
Kaempferol	Y = 0.1789x + 53.997	R^2^ = 0.9991	X = 10–110

The linearity plotted at 370 nm; y was the peak area; x was analytes; concentration μg·mL^−1^.

## 3. Experimental

### 3.1. Reagents and Materials

In this paper three flavonoids myricetin, quercetin and kaempferol were bought from the National Institute for the Control of Pharmaceutical and Biological Products; their batch numbers were 0709–9803, 100081–200406, 110861–200808, respectively. All ionic liquids were purchased from Shanghai Cheng Jie Chemical Co. Ltd (Shanghai, China). All chemical solvents were at least of analytical reagent grade, Ultrapure grade water (18MΩ, Millipore, Bedford, MA, USA) was used. HPLC grade acetonitrile used for HPLC analysis was purchased from Merck (Darmstadt, Germany). Identification of *Bauhinia championii (Benth.) Benth*. was confirmed by Professor Lu Wei, Fujian University of Traditional Chinese Medicine. Rattans of *Bauhinia championii (Benth.) Benth*. were dried, milled, passed through a stainless steel sieve and stored until use. The same batch of rattan was used through this study.

### 3.2. Flavonoid Extractions from Bauhinia Championii (Benth.) Benth

#### 3.2.1. Ionic Liquid-Based Microwave-Assisted Extraction (IL-MAE)

Ionic liquid-based microwave-assisted extraction (IL-MAE) was performed in a 2450 MHz MAE system with cold water running through the condenser (Sineo Microwave Chemistry Technology Company, Shanghai, China). Its maximum output power was 800 W and the power and temperature can be controlled with feedback/control video display. Temperature was measured by non-contact infrared measurement. Five g of dried rattan was mixed with different ILs (100 mL) with 0.8 mol/L HCl in a 250 mL quartz flat bottomed flask and then extracted by MAE at 70 °C for 10 min in open vessel mode. The optimum ILs, concentration of selected IL, sample particle size, microwave temperature, irradiation time, and solid-liquid ratio were systematically studied in this work. After extraction, the extracts were cooled down to room temperature, then filtrated and concentrated. Then the fraction is obtained by adding water and extracting using ethyl acetate. Finally, it was concentrated and reserved for further use. All extraction experiments were repeated three times.

#### 3.2.2. Conventional Reference Extraction Method

*Bauhinia championii (Benth.)* *Benth. *is pulverized to a coarse powder. Then it was refluxed with 70% (w/w) ethanol with 0.8 mol/L HCl for 1.5 h. The extraction solution is mixed and filtered. The filtered solution is concentrated to no ethanol taste. Then the ethyl acetate fraction is obtained by adding water and extracting using ethyl acetate. Finally, it was concentrated and reserved for further use. 

#### 3.2.3. Microwave-Assisted Extraction (MAE)

Rattan (5 g) was mixed with 70% ethanol with 0.8 mol/L HCl (100 mL) and then extracted by MAE under the same conditions used for IL-MAE. After extracting, the extracts were cooled down to room temperature, then were filtered and concentrated. Then the ethyl acetate fraction is obtained by adding water and extracting using ethyl acetate. Finally, it was concentrated and reserved for further use.

### 3.3. HPLC Analysis

The obtained samples were all filtered through a 0.45 μm microporous membrane before HPLC analysis on a DIONEX Ultimate 3000 liquid chromatography equipped with DAD detector (Dionex China Ltd., Shanghai, China). The mobile phase was consisted of: (A) acetonitrile and (B) 0.05% acetic acid solution (acetic acid-water = 0.05:100, v/v). The gradient elution program was as follows: 20% to 30% of solvent A from 0 to 5 min, then 30% to 50% of solvent A from 5 to 10 min, and held for 20 min. The other conditions of HPLC were: injection volume 20 μL, flow rate of 1 mL/min, the maximum absorption wavelengh was set at 370 nm and the column temperature kept at 30 °C. All experiments were done in triplicate. The yields of myricetin , quercetin and kaempferol were determined as follows:

Yield (mg/g) = Mass of myricetin (quercetin or kaempferol) (mg) in extraction solution (mg)/mass of sample (g)

### 3.4. LC-MS-MS Identification

An Agilent 6400 series Triple Quad (QQQ) LC-MS (Agilent, Beijing, China) instrument equipped with an electrospray ionization source (ESI) was employed to analyse the composition of fractions. The optimised detection parameters were as follows: Chromatographic conditions：the mobile phase A was 0.1% acetic acid solution, the mobile phase B was acetonitrile and the gradient elution conditions are shown in Table 3. Full wavelength scanning was from 190 to 400 nm, the flow rate was 0.4 mL/min, sampling volume was 5 μL, column temperature was 30 °C. Mass spectrometry conditions: negative ion mode. atomization gas pressure 40 psi, dry gas velocity 9 L/min, drying temperature degrees 350 °C，Ionization voltage 3,000 V, electrospray ionization (ESI), detection of anion way, auto MS^n^, scanning range 200~800 m/z.

**Table 3:** The chromatographic gradient elution conditions of the LC-MS analysis.

t/min	A	B
0	30	70
1.8	10	90
2.5	10	90
5.01	30	70

### 3.5. Statistical Methods

All the data are expressed by mean ± SD and use SPSS16.0 application of statistical software to do the statistical analysis. Student's t-test (SPSS Software Products, Chicago, IL, USA) were used to determine signiﬁcant differences between groups. *p* < 0.05 was accepted as statistically significant.

## 4. Conclusions

In this work, an IL-MAE approach was proposed to extract and quantify flavonoids from *Bauhinia championii (Benth.) Benth.* The effect of IL species and concentration was studied, as well as the optimum IL-MAE conditions. Compared with the HRE and regular MAE extraction methods, the proposed ILsMAE approach showed higher extraction efficiency and dramatically shorter extraction times (from 1.5 h to 10 min) under the optimum IL-MAE conditions of 2.0 M [bmim]Br solution with 0.80 mol/L HCl, a ratio of liquid to material at 30:1, and the extraction for 10 min at 70 °C. Under these optimal conditions, the yields of myricetin, quercetin and kaempferol were 0.0381 mg/g, 0.1352 mg/g, and 0.0474 mg/g. The crude extract could be used as either components of some complex traditional medicines or for further isolation and purification of the individual flavonoids. The results obtained are helpful in the utilization of *Bauhinia championii (Benth.)*. It also indicated that the IL-MAE approach is a green, simple, rapid, highly efficient extraction method for extracting important bioactive compounds from plant materials.
